# An Entomological Investigation during a Recent Rift Valley Fever Epizootic/Epidemic Reveals New Aspects of the Vectorial Transmission of the Virus in Madagascar

**DOI:** 10.3390/pathogens13030258

**Published:** 2024-03-16

**Authors:** Luciano Michaël Tantely, Soa Fy Andriamandimby, Maminirina Fidelis Ambinintsoa, Manou Rominah Raharinirina, Jean Théophile Rafisandratantsoa, Jean-Pierre Ravalohery, Aina Harimanana, Nirina Nantenaina Ranoelison, Judickaelle Irinantenaina, Miamina Fidy Ankasitrahana, Dany Bakoly Ranoaritiana, Laurence Randrianasolo, Rindra Vatosoa Randremanana, Vincent Lacoste, Philippe Dussart, Romain Girod

**Affiliations:** 1 Medical Entomology Unit, Institut Pasteur de Madagascar, Antananarivo 101, Madagascar; nihrii2202@gmail.com (M.F.A.); rmanourominah@pasteur.mg (M.R.R.); rgirod@pasteur.mg (R.G.); 2 Virology Unit, Institut Pasteur de Madagascar, Antananarivo 101, Madagascar; soafy@pasteur.mg (S.F.A.); theo@pasteur.mg (J.T.R.); jpierre@pasteur.mg (J.-P.R.); vlacoste@pasteur.mg (V.L.); pdussart@pasteur.mg (P.D.); 3 Epidemiology and Clinical Research Unit, Institut Pasteur de Madagascar, Antananarivo 101, Madagascar; aharim@pasteur.mg (A.H.); nranoelison@pasteur.mg (N.N.R.); judi@pasteur.mg (J.I.); laurence@pasteur.mg (L.R.); rrandrem@pasteur.mg (R.V.R.); 4 Direction de la Veille Sanitaire, de la Surveillance Epidémiologique et Ripostes, Ministry of Public Health, Antananarivo 101, Madagascar; fiidyankas@gmail.com (M.F.A.); rdanytia2@gmail.com (D.B.R.)

**Keywords:** Rift Valley fever, mosquito biology, outbreak, virus detection, Madagascar

## Abstract

A Rift Valley fever (RVF) outbreak occurred in at least five regions of Madagascar in 2021. The aim of this study was to provide an overview of the richness, abundance, ecology, and trophic preferences of mosquitoes in the Mananjary district and to investigate the distribution of mosquitoes that were RT-PCR-positive for RVFV. Three localities were prospected from 26 April to 4 May 2021, using light traps, BG-Sentinel traps baited with an artificial human odor, Muirhead-Thomson pit traps, and indoor pyrethroid spray catches. A total of 2806 mosquitoes belonging to at least 26 species were collected. Of 512 monospecific pools of mosquitoes tested with real-time RT-PCR, RVFV was detected in 37 pools representing 10 mosquito species. The RVFV-positive species were as follows: *Aedes albopictus*, *Ae. argenteopunctatus*, *Anopheles coustani*, *An. gambiae s.l.*, *An. mascarensis*, *An. squamosus*/*cydippis*, *Culex antennatus*, *Cx. decens*, *Cx. Tritaeniorhynchus*, and *Uranotaenia* spp. Of the 450 tested engorged females, 78.7% had taken a blood meal on humans, 92.9% on cattle, and 71.6% had taken mixed (human–cattle) blood meals. This investigation suggests the potential role of mosquitoes in RVFV transmission within this epizootic/epidemic context and that the human populations at the three study sites were highly exposed to mosquitoes. Therefore, the use of impregnated mosquito nets as an appropriate prevention method is recommended.

## 1. Introduction

Rift Valley fever virus (RVFV) belongs to the *Phlebovirus* genus, *Phenuiviridae* family, within the Bunyavirales order. It was first isolated after several infections were observed in sheep during a hepatitis outbreak in Kenya almost a century ago [1]. This virus is transmitted mechanically via direct contact with the body fluids (blood, saliva, and/or nasal discharges) or infected tissues of animals or aborted ruminant fetuses. Aerosols of infected blood generated during ruminant abortion or animal slaughter can also be a source of virus transmission to humans [2,3]. This virus can also be transmitted to vertebrate hosts via the bites of infected mosquitoes [4]. For some species, the vertical transmission of RVFV from infected female mosquitoes to their offspring has been observed and is described as the principal means of RVFV maintenance in the field [5]. The epidemic/epizootic cycle involves domestic ruminants (cattle, sheep, and goat) and humans, who are considered dead-end hosts [6]. Wild ruminants are mainly involved in a sylvatic cycle [7]. Birds, reptiles, and amphibians are refractory, and livestock species, such as pigs and horses, are resistant to infection but could play a subordinate role in the RVFV transmission cycle [8,9,10].

The first evidence of the vector transmission of RVFV dates back to 1944, when the virus was isolated from wild mosquitoes of the *Eretmapodites* and *Aedes* genera [11]. Since then, several epidemics/epizootics have been observed in numerous countries on the African continent [12], the Arabian Peninsula [13], and the islands of the southwestern Indian Ocean, including Madagascar [14], the Comoros [15], and Mayotte [16]. The occurrence and spread of RVF outbreaks in these regions depend on the ecoclimatic facies (Arabian Peninsula, West Africa, and East Africa) and the composition of the vector fauna, mainly comprising mosquitoes belonging to the *Aedes* and *Culex* genera [17]. The common feature of RVFV outbreaks in West and East Africa is that they are generally observed during the rainy season, when potential vectors are abundant [18,19]. The movement of infected cattle between countries has also been associated with RVF outbreaks in West [20] and East Africa [21].

In Madagascar, the circulation of RVFV was first reported in 1979 following its isolation from mosquito pools (monospecific pools of *Mansonia uniformis*, as well as pools containing a mixture of *Anopheles coustani*/*An. fuscicolor*, *An. pauliani*/*An. squamosus*, *Coquillettidia grandidieri*/*Ma. uniformis*, *Culex simpsoni*/*Cx. vansomereni*/*Cx. univittatus*, *Cx. antennatus*/*Cx. simpsoni*/*Cx. vansomereni*, and *Cx. simpsoni*/*Cx. vansomereni*/*Cx. annulioris*) captured in the primary rainforest of Périnet in the east of the country [22]. At that time, no circulation of the virus had been demonstrated in cattle or humans. Between 1982 and 1989, serological surveys of 824 human and 882 animal sera carried out in different regions showed little maintenance of the virus [23]. Then, two epizootic foci were observed in cattle in 1990 and 1991 on the east coast and in the Central Highlands, respectively, but the virus was not isolated from any mosquitoes [24,25]. From 1996 to 1998, serological investigations also showed the low-level circulation of the virus in herds, and no attempt has been made to detect nor isolate this virus from mosquitoes [26]. Ten years later, in 2006, the World Organization for Animal Health [27] confirmed that the virus was circulating on the island, with no clinical cases of RVF observed [28]. In 2008, the Institut Pasteur de Madagascar (IPM) confirmed the infection of 59 patients with RVFV, while the Ministry of Public Health (MoPH) recorded 17 deaths from 418 suspected cases [29]. From 2008 to 2009, RVF cases were recorded in humans and cattle in the northern, central, and southeastern parts of the country [30]. The virus was detected in three species of mosquitoes (*Cx. Antennatus*, *An. Squamosus*, and *An. coustani*) captured in the districts of Fianarantsoa I and II [31]. Retrospective studies have shown viral circulation in cattle in almost all Malagasy districts [32]. Since this last outbreak, no circulation of RVFV has been mentioned in livestock, but the research-based literature has highlighted the presence in the country of 24 mosquito species known to be associated with RVFV transmission [17]. Seven years after the 2008–2009 epidemic, three mosquito species (*Cx. antennatus*, *Cx. decens*, and *Cx. tritaeniorhynchus*), collected in the west of the country during the 2016 dry season, tested positive for RVFV [33], bringing the number of species potentially associated with RVFV transmission (*Cx. decens* being newly included in the list) to 25. In addition, a vector competence study of locally collected *Cx. antennatus* and *An. coustani* supported the role of both species in RVFV transmission in Madagascar [34].

On 2 April 2021, an epizootic of RVF in cattle in at least five regions of Madagascar (Atsimo–Andrefana, Vatovavy–Fitovinany, DIANA, Atsimo Atsinanana, and Alaotra Mangoro) was declared by the Ministry of Agriculture, Livestock and Fisheries (Ministère de l’Agriculture, de l’Elevage et de la Pêche, MAEP). The Vatovavy–Fitovinany region was the most affected. Two weeks later, on 15 April 2021, a suspected human case was identified at the Basic Health Center (Centre de Santé de Base, CSB) of Antsenavolo in the Mananjary health district of the Vatovavy–Fitovinany region. To assess the risk of transmission to humans and to propose appropriate prevention and vector control measures, a multidisciplinary investigation team, including entomologists, virologists, and epidemiologists of the IPM and the MoPH and MAEP, performed an investigation in the district of Mananjary with the aim of documenting the epidemiological, virological, and entomological situation (Supplementary Figure S1). The aim of this paper is to (i) describe the mosquito species composition, (ii) understand their ecology and assess their trophic preferences, and (iii) investigate the distribution of mosquitoes that are RT-PCR-positive for RVFV.

## 2. Materials and Methods

### 2.1. Study Sites

**Figure 1:**
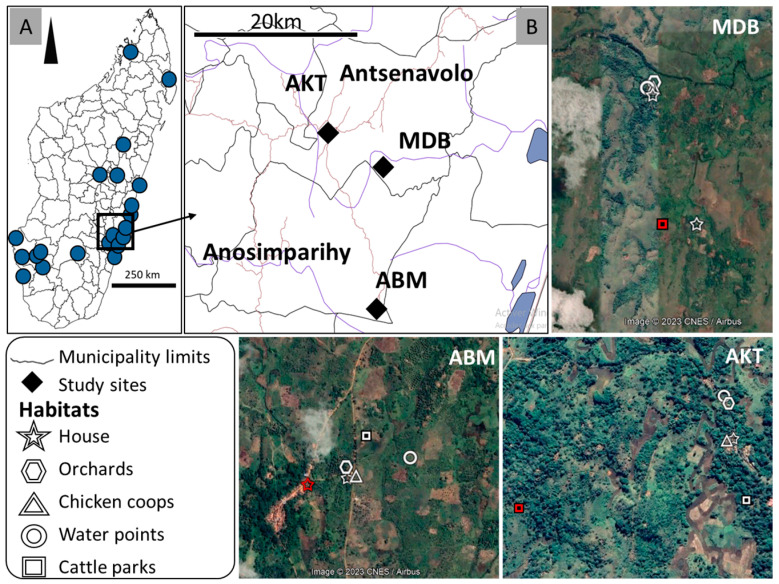
(**A**): The distribution of outbreaks of RVF in Madagascar, between 1 January 2021 and 25 November 2022, as reported to the WOAH through the early warning system [27]. (**B**): The locations of the three villages in the two municipalities of Antsenavolo and Anosimparihy and the five mosquito collection sites in each village. MDB: Madiolamba, ABM: Ambalamainty, and AKT: Ambodiakatra. A red star indicates a “house” habitat with a confirmed human case, and a red square indicates a “cattle park” habitat with a confirmed bovine case. The remaining habitats without confirmed human and bovine cases are white-colored.

Entomological investigations were carried out in three villages located in the municipalities of Antsenavolo and Anosimparihy in the district of Mananjary, in the Vatovavy region (Figure 1). The villages were chosen according to the presence of (i) suspected bovine and/or human cases known before the arrival onsite of the investigation team (municipality of Antsenavolo) and/or (ii) confirmed human cases even during the investigation (municipality of Anosimparihy).

The first village, Ambodiakatra (21°23′27.94″ S, 48°1′47.64″ E), is located along National Road 25, near the village of Antsenavolo, in the municipality of Antsenavolo. The landscape is made up of secondary forests with fruit trees (coffee, orange, mango, and lychee) and banana, coconut, and ravenale trees, as well as sugar cane fields. Rice fields are also located in small valleys separating the forested areas, and clove plantations are present near human habitations. Visible domestic animals are zebu, poultry, dogs, and pigs. Most of the farmers are also beekeepers. The second village, Madiolamba (21°25′6.79″ S, 48°6′13.62″ E), is more isolated, located 10 km southeast of the village of Antsenavolo, but still in the municipality of Antsenavolo. The landscape is similar, consisting of plantations of fruit trees, fields of sugar cane, and pineapples. The village is bordered by a stream, which makes the houses inaccessible during the rainy season when the waters rise. Rice fields are present in the small valleys separating the plantations of fruit trees. Domestic animals include zebu, poultry, dogs, and pigs. The third village, Ambalamainty (21°32′46.32″ S, 48°5′44.37″ E), is located 20 km southeast of the village of Antsenavolo, in the municipality of Anosimparihy. The landscape is similar. Rice fields, slash-and-burn crops, and swamps separate the plantations of fruit trees. The domestic animals present are zebu, poultry, and dogs. Beekeeping and clove cultivation are practiced by farmers.

### 2.2. Mosquito Sampling

From 26 April to 4 May 2021, adult mosquitoes were collected at each of the three villages during 48-h sessions by combining six light traps (LTs), which operated from 4:00 p.m. to 7:00 a.m.; six BG-Sentinel traps baited with an artificial human odor (BGs), which operated from 5:00 a.m. to 7:00 p.m.; and six Muirhead-Thomson pit traps (MTPTs) dug outside human dwellings. Mosquito collections were carried out with a mouth aspirator between 7:30 and 8:30 a.m. Furthermore, six sets of indoor pyrethroid spray catches (IPSCs) were carried out. Knocked-down mosquitoes were collected on white sheets between 7:30 and 8:30 a.m. The four methods (Supplementary Figure S1) were implemented in six habitat types (chicken coops, forest edges, orchards, water points, cattle parks, and houses where human and cattle RVF cases were suspected or confirmed). Forest edges and orchards were considered as a single habitat for subsequent analyses and are identified as “orchards” in the rest of the paper. 

### 2.3. Mosquito Identification and Storing

In the field, the adult mosquitoes collected were anesthetized with chloroform vapors and identified using the keys of Ravaonjanahary [35] for *Aedes*, Grjebine [36] for *Anopheles*, Doucet [37] for *Coquillettidia*, Edwards [38] for *Culex*, Brunhes and Hervy [39] for *Orthopodomyia*, and da Cunha Ramos and Brunhes [40] for *Uranotaenia* species. Immediately after morphological identification, the mosquitoes were counted; grouped into monospecific batches in cryotubes according to sex, the status of engorgement of females, the places and dates of collection, and the collection method used; and placed in liquid nitrogen. Upon return to the laboratory, they were stored at −80 °C pending further analysis.

### 2.4. Blood Meal Identification

The abdomens of engorged females were dissected and individualized in 2 mL microtubes. Total DNA was extracted using either a DNeasy Blood kit (Qiagen, Hilden, Germany) or QIAamp DNA kit (Qiagen). Samples were eluted in 200 µL of an AE Elution Buffer according to the manufacturer’s instructions and stored at −20 °C until use. The PCR technique developed by Pitzer et al. [41], with two forward primers designed to discriminate between blood meals taken from humans (Human-F: 5′-CTCGGCTTACTT CTCTTCC-3′) and zebu (Cattle-F: 5′-TTATCATCATAGCAATTGCC-3′), was used with a universal reverse primer (UnivRev: 5′-AGTGGGYGRAATATTATGC-3′) annealing at a conserved region for these two hosts.

The PCR mix was as follows: GoTaq Hot Start Green Master Mix (Promega, Madison, WI, USA) at a 1X final concentration, forward and reverse primers (at a 1 μM final concentration), and 2 μL of DNA diluted 1/10, in a final reaction volume of 25 μL. The PCR cycling conditions were as described by Pitzer et al. [41]. Amplicons were visualized on a 1.5% agarose gel.

### 2.5. RVFV Detection in Mosquitoes

Monospecific pools of 1 to 10 male mosquitoes, entire non-engorged females, or the heads and thoraxes of engorged females were created in 2 mL microtubes. The samples were then crushed in 200 µL of a cell culture medium (MEM) containing 40% fetal bovine serum, 2 mM L-glutamine, 1000 U/mL penicillin, 100 mg/mL streptomycin, and 2.5 mg/mL amphotericin B, for one minute, with 5 mm glass beads, using a TissueLyser II (Qiagen). After grinding, 200 µL of the ground material was transferred to a deep-96 well flat KF extraction plate. Viral RNA was extracted from the homogenates by using a KingFisher machine (Thermo Fisher Scientific, Waltham, MA, USA) according to the manufacturer’s instructions. Viral detection was performed via real-time RT-PCR using primers and a probe (RVF FP: 5′-TGCCAC GAGTYAGAGCCA-3′, RVF RP: 5′-GTGGGTCCGAGAGTYTGC-3′, and probe RVF P: (6FAM)-TCCTTCTCCCAGTCAGCCCCAC-(BHQ-1)) targeting the S segment of RVFV, as described by Weidmann et al. [42]. The limit of detection was determined to be 100 copies/reaction.

### 2.6. Statistical Analysis

All statistical analyses and most of the figures were generated using R version 4.2.2 [43]. For each mosquito genus and species, their abundance by village, type of habitat, and type of trap was compared using a Kruskal–Wallis test, followed by Dunn’s post hoc test to determine which pairs of groups were different. An Excel^®^ heatmap was used to visualize the variations in mosquito abundance between the three villages, the five habitat types, and the collected species. For blood meal analysis, a paired differential analysis between the human and cattle blood index of each species was obtained using the ggplot2 and ggpubr packages. Wilcoxon matched-pair signed-rank tests were used to compare paired human and blood indices. By using the ggalluvial packages in ggplot2, alluvial plots were generated to visualize the change in the number of engorged females collected across mosquito species, types of blood meals, habitat types, and sites.

## 3. Results

### 3.1. Mosquito Species Distribution and Abundance

Over six days, a total of 2806 mosquitoes were collected. Eleven genera and at least twenty-six species were identified, which were distributed as follows: *Aedeomyia* (one species), *Aedes* (at least three species), *Anopheles* (eight species), *Culex* (at least seven species), *Coquillettidia* (one species), *Eretmapodites* (one species), *Lutzia* (one species), *Mansonia* (one species), *Ficalbia* (at least one species), *Orthopodomyia* (one species), and *Uranotaenia* (at least one species). Four specimens could not be assigned to a genus (Table 1). Four species endemic to Madagascar (*Or. milloti*, *An. pauliani*, *Cq. grandidieri*, and *Cx. giganteus*) and one species endemic to Madagascar and the Comoros archipelago (*An. mascarensis*) were collected.

**Figure 2:**
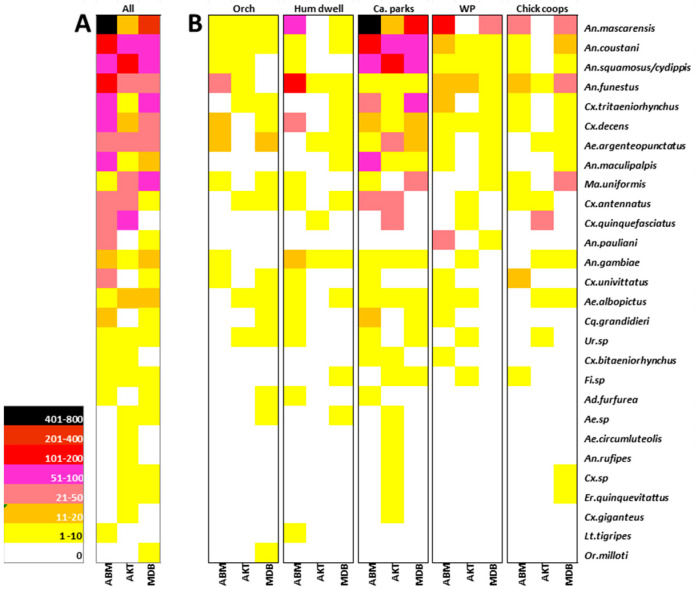
The abundance of mosquito species (in rows) collected by (**A**) site (ABM: Ambalamainty, AKT: Ambodiakatra, and MDB: Madiolamba) and (**B**) biotope (Orch: orchards and forest edges, Hum dwell: human dwellings, Ca. parks: cattle parks, WP: water points, and Chick coops: chicken coops).

The number of mosquitoes collected varied significantly according to the method used (Kruskal–Wallis X^2^ = 59.35, df = 3, *p* < 0.001). Mosquitoes were mainly collected using LTs (2638 mosquitoes, 26 species), with the exception of *Cx. Quinquefasciatus*, which was mainly collected with MTPs, and *Ae. albopictus*, with BGs. In terms of abundance, light trap collections were followed by IPSCs (76 mosquitoes, 5 species), BGs (53 mosquitoes, 10 species), and finally MTPTs (39 mosquitoes, 9 species) (Figure 2).

The genus *Anopheles* accounted for 74.3% (2806) of all collections, followed by the genus *Culex* (16.6%, 467). The other nine genera accounted for 9.0% (253) of the total. The number of mosquitoes collected varied significantly between species (Kruskal–Wallis X^2^ = 191.52, df = 25, *p* < 0.001).

*Anopheles mascarensis* was the most abundant species, accounting for 37.4% of all mosquitoes caught and 50.3% of all *Anopheles* collected. *Anopheles squamosus*/*cydippis* and *An. coustani* each accounted for 10.6% of all mosquitoes caught, *An. funestus* for 9.6%, *Culex tritaeniorhynchus* 6.0%, and *Cx. decens* 3.7%.

The number of mosquitoes collected varied significantly between the three villages (Kruskal–Wallis X^2^ = 44.481, df = 2, *p* < 0.001) and between the five habitats (Kruskal–Wallis X^2^ = 76.075, df = 4, *p* < 0.001) (Figure 2). The highest number of mosquitoes, with a total number of 1624 (57.9%), was collected in Ambalamainty, followed by the villages of Madiolamba (742, 26.4%) and Ambodiakatra (440, 15.7%). The predominance of *An. mascarensis* and *An. coustani* was particularly notable in Ambalamainty, while *An. squamosus*/*cydippis* was predominant in Ambodiakatra (Figure 2A). For the most abundant species, the majority of mosquitoes were caught near cattle parks (62.9%, 1764). Mosquitoes were more rarely collected at forest edges, regardless of the site (Figure 2B). The predominance of *An. gambiae*, *An. funestus*, and *Cx. decens* near human habitation was observed in Ambalamainty. Fourteen species were common to all three sites and a further ten to two of the three sites (Figure 2B).

Of the 26 species collected, 14 have already been reported to be involved in RVFV transmission [17]. They belonged to the *Anopheles*, *Aedes*, *Coquillettidia*, *Culex*, and *Mansonia* genera. Of these, *Anopheles coustani* (n = 299), *Anopheles squamosus*/*cydippis* (n = 297), *Culex tritaeniorhynchus* (n = 168), and *Culex decens* (n = 104) were the most abundant known vector species.

### 3.2. Blood Meal Analysis

**Figure 3:**
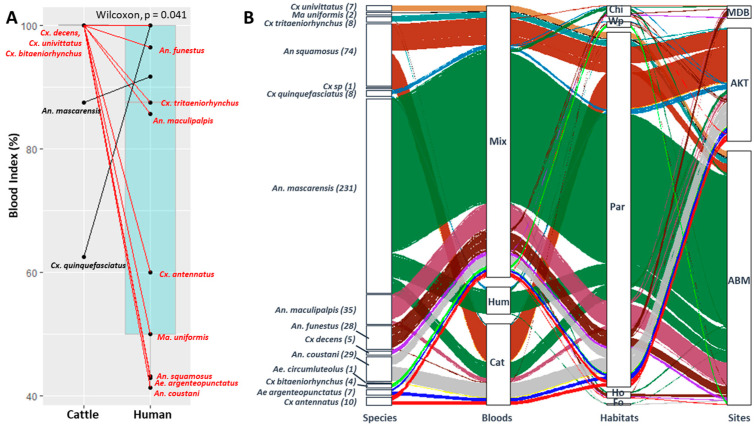
The analysis of blood meals. (**A**) The paired analysis of human and cattle blood indices (dots indicate the mosquito species; red lines represent the species that are predominantly zoophagic; and black lines indicate those that are predominantly anthropophagic). (**B**) Alluvial plots showing the changes in the number of engorged females for each species as a function of the type of blood meal (Cat: cattle, Hum: human, and Mix: mixed blood meals), habitat type (Fo: orchard, Ho: house, Par: cattle park, Wp: water point, and Chi: chicken coop), and site (ABM: Ambalamainty, AKT: Ambodiakatra, and MDB: Madiolamba). On the left-hand side of each block, the number of engorged females tested by species is indicated.

A total of 450 engorged females belonging to 14 species (plus one unidentified *Culex* sp.) were individually tested via PCR to determine the origins of their blood meals. Mosquitoes that had taken blood from cattle were significantly more numerous than those that had taken blood from humans (Wilcoxon test *p* < 0.05). Only *An. mascarensis* and *Cx. quinquefasciatus* had a greater percentage of blood meals taken from humans (Figure 3A).

Of the 450 engorged females, 301 were collected from ABM, 134 from AKT, and 15 from MDB (Figure 3B). Cattle parks provided 420 engorged females, followed by chicken coops (n = 13), human dwellings (n = 8), water points (n = 7), and forested habitats (n = 2). They were mostly collected via LTs (91.56%, n = 412), while 4.7% (n = 21) were from MTPTs, 3.11% (n = 14) from ISPCs, and only three from BGs.

Blood meals originating solely from humans or cattle were identified from 32 (7.1%) and 96 (21.3%) engorged females, respectively. Mixed blood meals, taken from humans and cattle, were identified from 322 (71.6%) engorged females. Hence, 92.9% of engorged females had taken a blood meal from cattle and 78.7% from humans. The three types of blood meals (human only, cattle only, and mixed) were only observed for *An. mascarensis* (Figure 3B).

### 3.3. Detection of RVFV in Mosquitoes

A total of 512 monospecific pools consisting of 2806 mosquitoes, including 249, 120, and 143 pools from ABM, AKT, and MDB, respectively, were tested via real-time RT-PCR. The viral genome was not detected in any of the 53 pools of male mosquitoes (for a total of 130 mosquitoes) (Table 2). Viral RNA was detected in 37 pools of female mosquitoes (n = 213), including 25 pools of non-engorged females (n = 152) and 12 pools of engorged females (n = 61), belonging to ten species (38.5% of the species). Thirteen positive pools were from ABM, 12 were from AKT, and the last 12 were from MDB (Table 2). The species that were found to be RVFV-positive were as follows: *Aedes albopictus* (1 out of 18 non-engorged female pools), *Ae. argenteopunctatus* (1 out of 18 non-engorged female pools and 2 out of 2 engorged female pools), *Anopheles coustani* (1 out of 35 non-engorged females pools and 2 out of 5 engorged females pools), *An. gambiae s.l.* (1 out of 12 non-engorged female pools), *An. mascarensis* (6 out of 92 non-engorged female pools and 3 out of 29 engorged female pools), *An. squamosus*/*cydippis* (6 out of 30 non-engorged female pools and 2 out of 11 engorged female pools), *Culex antennatus* (2 out of 15 non-engorged female pools and 2 out of 5 engorged female pools), *Cx. decens* (1 out of 17 non-engorged female pools and 1 out of 5 engorged female pools), *Cx. tritaeniorhynchus* (5 out of 24 non-engorged female pools), and *Uranotaenia* sp (one out of seven non-engorged female pools).

### 3.4. RVFV Detection and Blood Meal Sources

Twelve monospecific pools of engorged females from six species were found to be positive for RVFV. The two positive pools of *Ae. argenteopunctatus*, as well as two of *Cx. antennatus*, contained females that had taken a mixed meal or a meal on cattle exclusively (Table 3). Three pools of *An. mascarensis* contained females that had taken either a mixed meal or a meal on humans exclusively. The pools of *An. coustani*, *An. squamosus*/*cydippis*, and *Cx. decens* contained only females that had taken a mixed blood meal. In all, 74.6% (44/59) of engorged females from the RVFV-positive pools had taken a mixed blood meal (Table 3).

## 4. Discussion

The reappearance of RVF in Madagascar in 2021, twelve years after the second major outbreak reported on the island (2008–2009), showed the persistence of a recurrent substantial risk for the human population and livestock locally. It should be remembered that RVF epidemics/epizootics occur when the circulation of RVFV is amplified by mosquito populations in an environment favorable to their proliferation [44]. In this context, the entomological component was inventoried during a multidisciplinary investigation conducted in the Mananjary district of the Vatovavy–Fitovinany region from 26 April to 4 May 2021.

A total of 2806 mosquitoes belonging to at least 26 species were caught. Among them, 14 species were already known to be associated with RVFV transmission in Madagascar [17]. *Anopheles mascarensis* was the most abundant species, accounting for 37.4% of all mosquitoes caught. This species has never been reported to be infected with RVFV in the literature [17]. In addition, of the 2806 mosquitoes, 450 blood-fed females were caught. A mixed blood meal was taken by 71.6% of engorged females and three quarters of those from RVFV-positive pools. These results led us to identify the mosquito species likely to act as RVFV vectors at the three study sites during this epidemic.

### 4.1. Species Diversity and Abundance

Most mosquitoes (94.0% of total individuals) were caught with light traps. The use of LTs was based not only on their higher productivity as compared to other types of traps [23,45,46,47] but also on their suitability for the collection of nocturnal mosquito species involved in RVFV transmission [31]. This method is extensively used during RVFV outbreak investigation [31] and inter-epizootic periods [33].

The variation in mosquito abundance according to the type of trap, sampling site, and habitat type recorded in this study has already been reported in other studies [47,48,49]. The highest abundance of *Cx. quinquefasciatus* in Ambodiakatra, the most anthropized area, was expected due to the urban nature of the species [47]. With the exception of species belonging to the *Orthopodomyia*, *Uranotaenia*, and *Ficalbia* genera, the majority of the species collected in this study have been commonly collected in previous entomological studies on RVFV [31]. Notably, this is the first study to report the predominance of *Anopheles mascarensis*, raising questions about the role of this species in RVFV transmission and in the 2021 RVF epidemic.

Of the twenty-six-mosquito species collected, six (*An. squamosus*/*cydippis*, *An. coustani*, *Cx. antennatus*, *Cx. decens*, *Cx. tritaeniorhynchus*, and *Ma. uniformis*) were already found to be naturally infected with RVFV in Madagascar [23,31,33], six others (*Ae. circumluteolus*, *An. gambiae sl*, *Cx. bitaeniorhynchus*, *Cx. quinquefasciatus*, *Cx. univittatus*, and *Er. quinquevittatus*) were found in other countries, and two species (*Ae. albopictus* and *Ae. argenteopunctatus*) had known vector competence [17].

### 4.2. Trophic Behavior

A second condition necessary for viral circulation and epidemic emergence is the meeting between a virus and its vertebrate host [50], probably during mosquito feeding. In the case of RVFV, the trophic preferences of the different mosquito species towards domestic ruminants and humans are a determining element. In the present study, PCR primers allowing for discrimination between human and cattle blood meals were used based on the involvement of these two hosts in the RVFV epidemiological cycle in Madagascar [12]. With the exception of *Ae. circumluteolus*, all of the mosquito species shown to feed on cattle in the present study were shown to feed on humans too, supporting previous studies that these species are opportunistic feeders [47,51]; however, although *An. mascarensis* and *Cx. quinquefasciatus* were shown to feed on cattle, they were shown to be mostly anthropophilic, as already reported [47,52]. For *An. mascarensis*, two sibling species (one mostly anthropophilic and other mostly zoophilic species) have been reported in Madagascar [52]. It is possible that this anthropophilic *An. mascarensis* population is one sibling species of this species, as already mentioned by Fontenille [52].

The high number of mixed blood meals (more than 70%) observed here was in line with that reported in host-feeding pattern studies of culicine mosquitoes, allowing for the explanation of the 2006–2007 Kenyan RVFV outbreak [53] and regular dengue, as well as malaria outbreaks in Thailand [54].

### 4.3. Virus Detection

Another necessary condition for viral circulation and the emergence of an epidemic is the “compatibility” of a vector to a virus [50]. Tantely et al. [17] proposed the classification of mosquito vectors according to three criteria: natural infection, vector competence, and vector–host contact in the field. Based on these criteria, three species were validated as being major vectors of RVFV: two as candidate vectors and one as a potential vector in Madagascar (Table 2). In the present study, ten species tested positive for RVFV. The repeated detection of RVFV in *An. squamosus*, *An. coustani*, *Cx antennatus*, *Cx. decens*, and *Cx. tritaeniorhynchus* in Madagascar supports the full incrimination of these species as vectors according to Hamon et al. [55].

*An. squamosus*/*cydippis*: *An. squamosus* and *An. cydippis* are morphologically identical and indistinguishable from each other. While *Anopheles cydippis* has never been implicated in pathogen transmission, *Anopheles squamosus* was found to be positive for RVFV during the 2008–2009 epidemic [31]. This species is known for its zoo-anthropophilic behavior [36,45]. In this study, 43.2% of the blood meals identified from this species were mixed blood meals taken from cattle and humans. The detection of RVFV in this species and its abundance, particularly near cattle parks, suggest its role as a candidate vector in the RVF epizootic/epidemic in the Mananjary district. Vectorial competence is the only missing information for its consideration as a major vector.

*An. coustani*: Ratovonjato et al. [31] were the first to identify *An. coustani* as being naturally infected with RVFV in Madagascar, while this species was already found to be naturally infected with RVFV in mainland Africa [56]. This species is mostly zoophilic, and no information is available on its ability to transmit RVFV. Based only on the experimental infection rates reported in the literature [34], not enough information is available to evaluate this species as a major RVFV vector.

*Cx. antennatus*: This species is zoo-anthropophilic [51]. RVFV was isolated from it during the 2009 epidemic/epizootic in the districts of Fianarantsoa I and Ambalavao [31], as well as in 2016, during the inter-epizootic period, in the Ambatoboeny district [33]. During this study, *Cx. antennatus* collections were significant at the Ambalamainty and Ambodiakatra sites, particularly near the zebu parks. In the literature, this species has been reported to be susceptible to RVFV infection and capable of transmitting it [4,34,57]. All these observations confirm its role as a major vector of RVFV [17].

*Cx. decens*: This species can exhibit anthropophilic behavior [23] and may be attracted to livestock and poultry [45]. The RVFV genome was detected from *Cx. decens* in 2016 in the Ambatoboeny and Anivorano districts in the north of the country [33]. Significant densities of this species were observed during the present survey, but, given that no information is available on its potential to transmit RVFV, *Cx. decens* remains a candidate vector for RVFV.

*Cx. tritaeniorhynchus*: The species has already been found to be PCR-positive for RVFV in the Ambatoboeny district in 2016 [33] and was reported as experimentally susceptible to RVFV infection [58]. The zoo-anthropophilic nature of this species is confirmed by the high proportion of females (87.5%) with mixed blood meals obtained during this study. *Cx. tritaeniorhynchus* can therefore be considered an RVFV candidate vector pending information on its potential to transmit RVFV.

This study is the first to report the RT-PCR positivity of *An. gambiae sl.*, *Aedes albopictus*, *Ae. argenteopunctuatus*, and an unidentified *Uranotaenia* sp. for RVFV in Madagascar.

*An. gambiae sl*: Three species of this complex are present in Madagascar: *Anopheles gambiae*, *An. arabiensis*, and *An. merus* [59]. RVFV was detected via RT-PCR from wild-caught populations of *An. arabiensis* in Sudan [60]. In addition, RVFV infection and dissemination were also reported from laboratory colonies of *An. gambiae* [61,62,63,64].

*Ae. albopictus*: To our knowledge, this is the first report of *Ae. albopictus* testing positive via RT-PCR for RVFV, although RVFV infection and transmission have been reported from laboratory colonies [62]. This species is diurnal and highly anthropophilic [23]. The three criteria that make this species a major vector are met; however, the highly anthropophilic behavior of this species [23] should not lead to new outbreaks due to the fact that humans are considered dead-end hosts [65]. Nonetheless, RVFV maintenance by *Ae. albopictus* via vertical transmission, as already suggested in Madagascar [17], should not be neglected.

*Ae. argenteopunctatus*: This study is also the first to mention the detection of RVFV via RT-PCR in *Ae. argenteopunctatus*. It is an anthropophilic species [23] reported to exhibit zoophilic behavior by feeding on domestic ruminants in Africa [66]. RVFV transmission was also reported from laboratory colonies [63]. This species belongs to the *Aedimorphus* subgenus, for which RVFV vertical transmission has already been suggested [67]. The three criteria that make this species a major vector are met, but this species should exploit this major vector role locally (where this species occurs in abundance).

*Uranotaenia*: This is the first study to report a mosquito belonging to the genus *Uranotaenia* as being RT-PCR-positive for RVFV, but this genus was already found to harbor a Phlebovirus in the Ivory Coast [64]. With the information obtained in this study, the *Uranotaenia* genus should be considered as a potential vector for RVFV.

*An. mascarensis*: Among the ten species found to be RT-PCR-positive for RVFV, *An. mascarensis* deserves special attention. This species, endemic to Madagascar and the Comoros archipelago, was already found to be infected with *Plasmodium* [52], Ngary virus [23], and *Wuchereria bancrofti* [68], but has never been associated with RVFV transmission. In the present study, RVFV was detected in six out of nine pools of non-engorged females, suggesting that RVFV had already disseminated [49]. The detection of RVFV via RT-PCR in wild-caught populations of *An. mascarensis* suggests that this species could be susceptible to the virus [57]. Unfortunately, no information is available on its ability to transmit it. Although this species is abundant and displays highly zoo-anthropophilic behavior, its role as an RVFV candidate vector remains to be further explored through viral isolation and experimental infection attempts.

This species is reputed to be a vector of *Plasmodium* parasites in the country [69], and the Mananjary district is located in an area of high and stable malaria transmission [70]. The co-infection of *An. mascarensis* with *Plasmodium* parasites could facilitate RVFV transmission due to the disruption of the salivary glands [71]. Furthermore, although *An. mascarensis* is assumed to be an incompetent vector of RVFV, the high abundance and zoo-anthropophilic behavior of this species could favor RVFV outbreaks. Indeed, according to yellow fever’s epidemiology [72], high population densities, such as those observed here, of an incompetent mosquito vector could initiate and maintain viral transmission.

### 4.4. Implications for RVFV Transmission to Humans and Animals and Recommendations

This multidisciplinary investigation allowed for the identification of the occurrence of mosquitoes, vertebrate hosts, and RVFV in the same place and at the same time, thus meeting the first necessary condition for the circulation of the virus and the emergence of RVF in livestock and humans. Entomologically, there are now eleven species that have been found to be RT-PCR-positive for RVFV from wild-caught populations in Madagascar, with *An. gambiae sl.*, *An. mascarensis*, *Ae. albopictus*, *Ae. argenteopunctuatus*, and an unidentified species of *Uranotaenia* newly included in the list.

By combining the 24 mosquito species potentially associated with RVFV transmission listed by Tantely et al. [17], plus the two species (*Cx. decens* and *Cx. tritaeniorhynchus*) found to be RT-PCR-positive for RVFV in 2016 in the western region of Madagascar [33] and *An. mascarensis*, there are now 27 species associated with RVFV transmission in Madagascar [17].

Compared with previous RVFV epidemics/epizootics [14,24,30,32], the 2021 outbreak was the largest epizootic ever recorded in the country [27]. Cattle movements within the island, as observed in Mananjary, are a possible cause of this widespread RVFV outbreak [14,24,73]. In addition, we observed that cattle were an important blood meal source for all mosquito species, suggesting that they could have served as virus-amplifying hosts for mosquito species able to transmit RVFV [7]. Indeed, susceptible cattle rapidly develop high-titer viremia (1.0 × 10^2^ PFUs/mL of blood) two days post-infection [74], and viremia higher than 10^1.3^ PFUs/mL is enough to infect mosquito vectors [62].

The larval stages of mosquitoes associated with RVFV can develop in streams, lakes, ponds, marshes, banks, backwaters, irrigation drains, nursery waters, brackish waters, lagoons, wet ponds, and rice fields [23,51]. The configuration of the larval breeding habitats of these species makes it difficult to envisage the application of larval control measures. As the mosquito species involved in RVFV transmission are nocturnal [17], the use of impregnated mosquito nets appears relevant to reduce exposure to endophagic and endophilic mosquitoes; however, the application of indoor vector control measures (the use of impregnated mosquito nets and the indoor spraying of residual insecticides) is of limited effectiveness, due to the fact that all species found to be positive for RVFV also exhibit exophagic and exophilic behavior [48], except *Uranotaenia*, with unknown behavior.

Fortunately, the distribution of mosquito nets impregnated with long-acting insecticides is already planned as part of the national malaria control program in the Mananjary district. Indeed, some of the species in question (*An. mascarensis* and *An. gambiae sl*) are also known to transmit malaria parasites. Moreover, the implementation of indoor spraying operations might not be accepted by villagers, in an attempt to avoid an undesirable effect on their beekeeping activity.

## 5. Conclusions

The abundance, diversity of species, origin of blood meals, and identification of RVFV in some monospecific pools of mosquitoes enable the incrimination of mosquito vectors in the transmission of RVFV during this epidemic/epizootic. *Anopheles mascarensis* could initiate an RVFV outbreak, and this species, with the remaining nine RVFV-positive species, may have acted as bridge vectors for the virus, contributing to the high numbers of infected animals and humans [75,76]. In addition, with the high number of mixed blood meals observed, high numbers of human cases were expected; however, few careful clinical observations are generally reported in African countries. In any case, with the close association of humans with domestic animals being an important risk factor for infection, important epidemics are expected after epizootic activity [77]. Hitherto, the long inter-epizootic periods, of 12 to 17 years, stated for the country [45] are still highlighted, and the detection of RVFV in mosquitoes (*Cx. decens*, *Cx. tritaeniorhynchus*, and *Cx. antennatus*) from the northwest region in 2016 [33] supports the existence of low-noise viral circulation in livestock during inter-epizootic periods [22,23,25,26,30]. The high percentage of blood meals taken on humans shows that the human populations at the three investigated study sites in the Mananjary district were highly exposed to potentially infectious mosquito bites. Nevertheless, vector competence experiments should be performed to confirm that these mosquito species can transmit the virus. Promoting the use of impregnated mosquito nets appears to be the most appropriate prevention method in order to limit the transmission of RVFV in the human population of the Mananjary district.

## Figures and Tables

**Table 1:** The distribution of adult mosquitoes collected by species, collection site, and collection method.

Species	Ambalamainty				Ambodiakatra				Madiolamba				Total
	BGs	LTs	MTPTs	IPSCs	BGs	LTs	MTPTs	IPSCs	BGs	LTs	MTPTs	IPSCs	
*Ad. furfurea*	0	2	0	0	0	0	0	0	0	1	0	0	3
*Ae. albopictus* *	7	0	0	0	8	0	1	2	6	5	0	0	29
*Ae. argenteopunctatus* *	0	23	0	0	0	31	1	0	0	36	0	0	91
*Ae. circumluteolus* ^£, V^	0	0	0	0	0	2	0	0	0	0	0	0	2
*Ae.* sp.	0	0	0	0	0	1	0	0	0	2	0	0	3
***An. coustani*** **^, V^	0	143	0	0	0	69	0	0	0	87	0	0	299
*An. funestus*	0	190	6	4	1	17	2	6	0	33	1	9	269
*An. gambiae sl* ^£, V^	1	13	0	0	0	8	0	1	0	11	0	0	34
*An. maculipalpis*	0	64	0	0	0	7	0	0	2	12	0	0	85
*An. mascarensis*	5	765	18	0	0	18	0	0	0	241	2	0	1049
*An. pauliani*	0	50	0	0	0	0	0	0	0	1	0	0	51
*An. rufipes*	0	0	0	0	0	2	0	0	0	0	0	0	2
***An. squamosus*/*cydippis*** **	0	91	0	0	0	123	0	0	0	83	0	0	297
*Cq. grandidieri*	0	14	0	0	0	0	0	0	0	5	0	0	19
***Cx. antennatus*** ***	0	44	0	0	0	26	4	1	1	2	0	0	78
*Cx. bitaeniorhynchus* ^£^	0	5	0	0	0	0	1	0	0	0	0	0	6
***Cx. decens*** **	0	64	0	0	0	12	0	0	0	27	1	0	104
*Cx. giganteus*	0	0	0	0	0	1	0	0	0	0	0	0	1
*Cx. quinquefasciatus* **^, £^	0	0	0	0	17	4	1	53	0	0	0	0	75
***Cx. tritaeniorhynchus*** ***	2	63	0	0	0	10	0	0	0	93	0	0	168
*Cx. univittatus* **^, £, V^	0	32	0	0	0	0	0	0	0	1	0	0	33
*Cx.* sp.	0	0	0	0	0	1	0	0	0	1	0	0	2
*Er. quinquevitattus* **^, £^	0	0	0	0	1	0	0	0	0	1	0	0	2
*Ficalbia* sp.	0	1	0	0	0	3	0	0	0	2	0	0	6
***Ma. uniformis*** **	0	9	0	0	0	0	0	0	0	73	0	0	82
*Or. milloti*	0	0	0	0	0	0	0	0	0	1	0	0	1
*Ur.* sp.	0	4	0	0	1	3	0	0	0	1	1	0	10
*Lt tigripes*	0	1	0	0	0	0	0	0	0	0	0	0	1
*Unidentified genus*	1	2	0	0	0	1	0	0	0	0	0	0	4
Total	16	1580	24	4	28	339	10	63	9	719	5	9	2806

In bold are species already found naturally infected with RVFV in Madagascar in monospecific batches. ^£^: species found infected with RVFV in other regions. ^V^: species capable of vertical transmission. BGs: BG-Sentinel traps baited with an artificial human odor. LTs: light traps. MTPTs: Muirhead-Thomson pit traps. IPSCs: indoor pyrethroid spray catches. Tantely et al. [17] proposed the classification of mosquito vectors according to three criteria, these being natural infection, vector competence, and field vector–host contact. These three criteria are validated in RVFV major vectors (***), two of them in candidate vectors (**), and one of them in potential vectors (*).

**Table 2:** The number of monospecific pools of mosquitoes tested and found positive via RT-PCR for RVFV by species, sex, the status of engorgement for females, and site.

	Ambalamainty			Ambodiakatra			Madiolamba			Total
	Females		Males	Females		Males	Females		Males	
Species	Go	Non-Go		Go	Non-Go		Go	Non-Go		
*Ad. furfurea*	-	2	-	-	-	-	-	1	-	3
***Ae. albopictus*** *** ^, µ^	-	5	1	-	6 (1)	1	-	7	1	21 (1)
***Ae. argenteopunctatus*** *** ^, µ^	-	4	2	2 (2)	6 (1)	-	-	8	-	22 (3)
*Ae. circumluteolus* ^£, V^	-	-	-	1	1	-	-	-	-	2
*Ae.* sp.	-	-	-	-	1	-	-	2	-	3
***An. coustani*** **^, V^	1 (1)	17 (1)	3	4 (1)	6	1	-	12	1	45 (3)
*An. funestus*	4	23	2	3	6	2	3	7	3	53
***An. gambiae sl*** **^, £, V, µ^	-	4	2	-	4	2	-	4 (1)	1	17 (1)
*An. maculipalpis*	3	6	-	1	1	-	1	4	-	16
***An. mascarensis*** **^, µ^	26 (3)	60	4	2	3 (1)	-	1	29 (5)	2	127 (9)
*An. pauliani*	-	5	-	-	-	-	-	1	-	6
*An. rufipes*	-	-	-	-	2	-	-	-	-	2
***An. squamosus*/*cydippis*** **	2	12 (3)	1	8 (2)	9 (2)	-	1	9(1)	-	42 (8)
*Coquillettidia grandidieri*	-	4	-	-	-	-	-	2	1	7
***Cx. antennatus*** ***	1 (1)	7 (1)	-	4 (1)	6 (1)	-	-	2	-	20 (4)
*Cx. bitaeniorhynchus* ^£^	1	1	-	1	-	-	-	-	-	3
***Cx. decens*** **	2 (1)	9 (1)	4	-	3	-	1	5	2	26 (2)
*Cx. giganteus*	-	-	-	-	1	-	-	-	-	1
*Cx. quinquefasciatus* **^, £^	-	-	-	3	6	10	-	-	-	19
***Cx. tritaeniorhynchus*** ***	1	9	-	2	3	-	-	12 (5)	1	28 (5)
*Cx. univittatus* **^, £, V^	1	6	2	-	-	-	-	1	-	10
*Cx.* sp.	-	-	-	1	-	-	-	1	-	2
*Er. quinquevitattus* **^, £^	-	-	-	-	-	1	-	1	-	2
*Ficalbia* sp.	-	1	-	-	2	1	-	2	-	6
*Ma. uniformis* **	1	5	-	-	-	-	-	10	1	17
*Or. milloti*	-	-	-	-	-	-	-	-	1	1
***Ur.* sp.** *^, µ^	-	2 (1)	-	-	3	-	-	2	-	7 (1)
*Lt. tigripes*	-	1	-	-	-	-	-	-	-	1
*Genre* sp.	-	2	-	-	1	-	-	-	-	3
Total	43 (6)	185 (7)	21	32 (6)	70 (6)	18	7	122 (12)	14	512 (37)

Go: number of monospecific pools of engorged females tested; Non-Go: number of monospecific pools of non-engorged females tested; and Males: number of monospecific pools of males tested. Between brackets: number of monospecific pools found positive via RT-PCR. In bold: species naturally infected with RVFV or RT-PCR-positive for RVFV in Madagascar. ^µ^: species newly found to be RT-PCR-positive for RVFV in Madagascar. ^£^: species found to be infected with RVFV in other regions. *** main vector, ** candidate vector, and * potential vector species. ^V^: species capable of vertical transmission.

**Table 3:** The origins of the blood meals taken by engorged female mosquitoes constituting the monospecific pools found to be positive for RVFV.

Species	Engorged Positives		Blood Meals (Mixed and Single)		
	RVFV + Pools	Ind Go/Pool+	Mixed	Human	Cattle
*Ae. argenteopunctatus*	2	4 * + 5	3	0	4
*An. coustani*	2	1 + 5	6	0	0
*An. mascarensis*	3	10 + 10 + 10	20	10	0
*An. squamosus*/*cydippis*	2	2 + 10	12	0	0
*Cx. antennatus*	2	1 + 2	2	0	1
*Cx. decens*	1	1	1	0	0

RVFV + pools: number of monospecific pools found to be positive for RVFV, Ind go/pool: number of engorged females per RVFV-positive pool, and *: two engorged females without an abdomen (origin of blood meal unknown).

## Data Availability

All data provided and analysed in this study are publicly available. Please contact Luciano Michaël Tantely.

## Supplementary Materials

The following supporting information can be downloaded at: https://www.mdpi.com/article/10.3390/pathogens13030258/s1, Supplementary Figure S1: Illustration of the multidisciplinary team performing an investigation in the district of Mananjary with a mobile laboratory in a four-wheel-drive vehicle used to perform the laboratory procedures in the field conditions. B: Field workers crossing a river to reach Madiolamba village. C: Field workers preparing and deploying mosquito traps in the village of Madiolamba. LT: light trap, IPSC: indoor pyrethroid spray catch; BG: BG-Sentinel trap, and MTPT: Muirhead-Thomson pit traps.
