# Supplementary figures and images for: An Entomological Investigation during a Recent Rift Valley Fever Epizootic/Epidemic Reveals New Aspects of the Vectorial Transmission of the Virus in Madagascar
Source: Pathogens. 2024 Mar 16;13(3):258. doi: 10.3390/pathogens13030258 (PMC10975538; doi:10.3390/pathogens13030258)

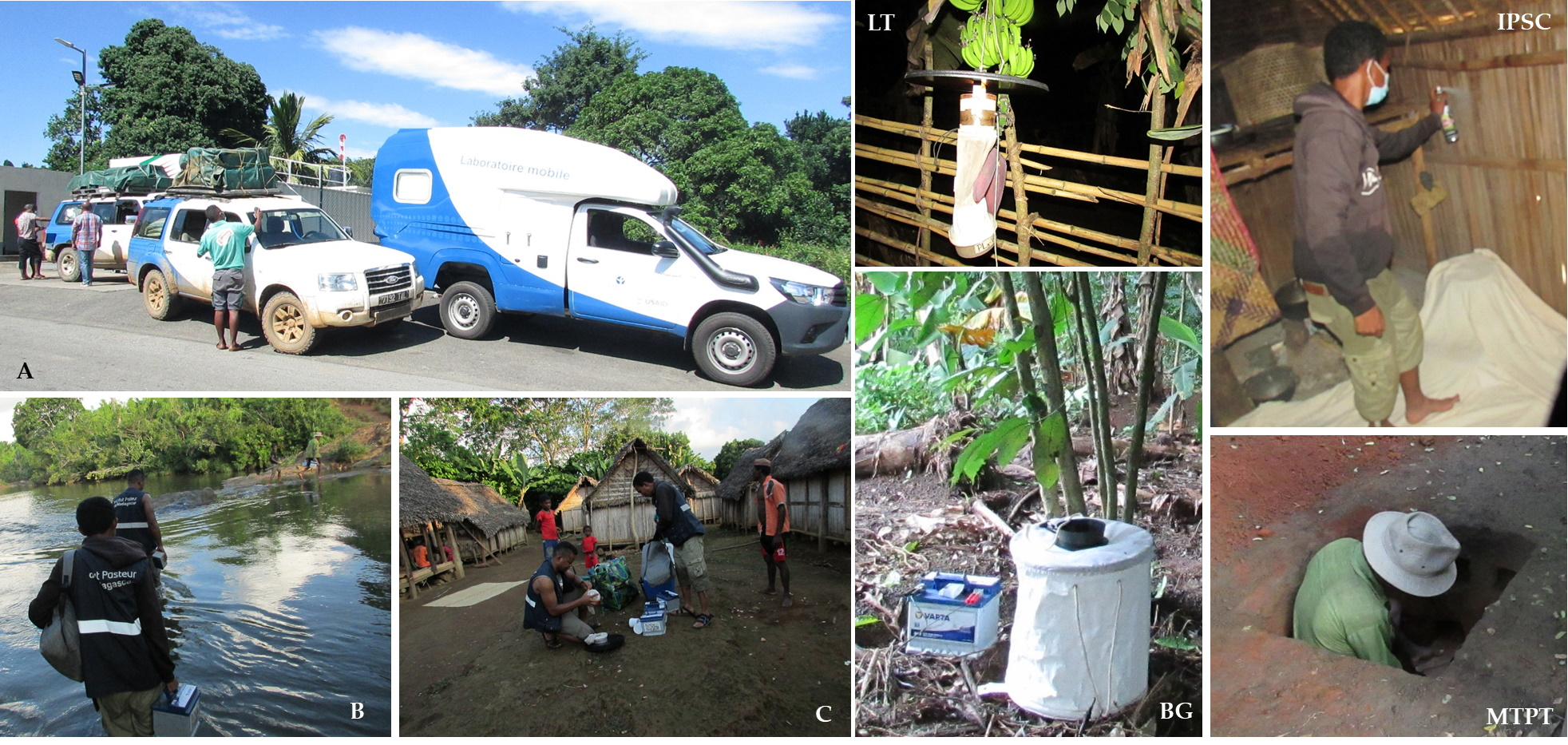

Supplement: Supplementary file 1 [file pathogens-13-00258-s001.zip › pathogens-2880458-supplementary.png]
